# Reply to “Comment on López-Yerena et al. ‘Absorption and Intestinal Metabolic Profile of Oleocanthal in Rats’ *Pharmaceutics* 2020, *12*, 134”

**DOI:** 10.3390/pharmaceutics12121221

**Published:** 2020-12-17

**Authors:** Anallely López-Yerena, Anna Vallverdú-Queralt, Raf Mols, Patrick Augustijns, Rosa M. Lamuela-Raventós, Elvira Escribano-Ferrer

**Affiliations:** 1 Nutrition, Food Science and Gastronomy Department, XaRTA, Institute of Nutrition and Food Safety (INSA-UB), School of Pharmacy and Food Sciences, University of Barcelona, 08028 Barcelona, Spain; naye.yerena@gmail.com (A.L.-Y.); avallverdu@ub.edu (A.V.-Q.); lamuela@ub.edu (R.M.L.-R.); 2 CIBER Physiopathology of Obesity and Nutrition (CIBEROBN), Institute of Health Carlos III, 28029 Madrid, Spain; 3 Drug Delivery and Disposition, KU Leuven, 3000 Leuven, Belgium; raf.mols@kuleuven.be (R.M.); patrick.augustijns@pharm.kuleuven.be (P.A.); 4 Biopharmaceutics and Pharmacokinetics Unit, Department of Pharmacy and Pharmaceutical Technology and Physical Chemistry, Institute of Nanoscience and Nanotechnology (IN2UB), Pharmacy and Food Sciences School, University of Barcelona, 08028 Barcelona, Spain

**Keywords:** bioavailability, metabolites, in vivo study, permeability, extra virgin olive oil

## Abstract

Recently, in February 2020, we published a study exploring the intestinal absorption and metabolism of oleocanthal (OLC) in rats. A single-pass intestinal perfusion technique (SPIP) was used, involving simultaneous sampling from the luminal perfusate and mesenteric blood. Later, comments on our published paper were released, requesting clarification of specific data. In this detailed reply, we hope to have addressed and clarified all the concerns of A. Kaddoumi and K. El Sayed and that the scientific community will benefit from both the study and the comments it has generated.

## 1. Introduction

We appreciate the interest of A. Kaddoumi and K. El Sayed in our study and thank them for providing us with the opportunity to make improvements and add some new information. The following is our response to their comments, which can be divided in two parts, one related to the oleocanthal (OLC) purity and analysis of the biological samples, and the other concerning the perfusion technique used in rats.

## 2. Reply to the Comments

### 2.1. Purity of Oleocanthal and Biological Sample Analysis

**Figure 1:**
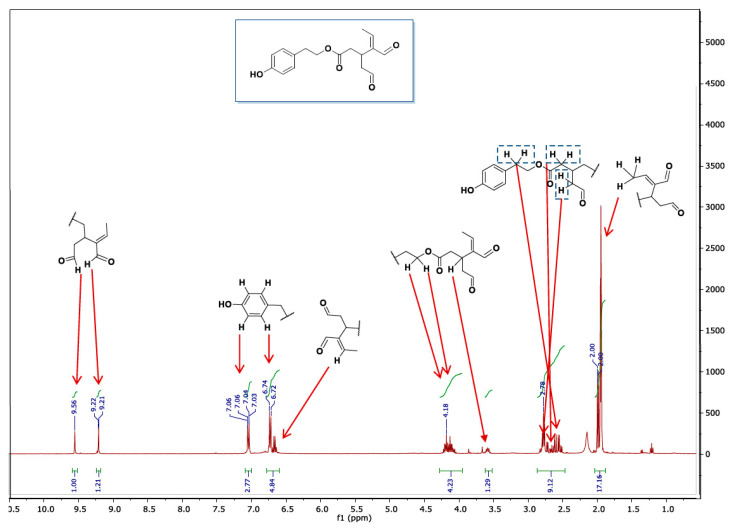
NMR spectrum of oleocanthal.

According to the commercial supplier, the OLC has a purity ≥ 90. However, to confirm this, although it is not described in the paper, the OLC was analyzed using ^1^H NMR prior to any of the experimental work (Figure 1).

As can be observed in the spectrum, the aromatic signals belonging to the protons H-8′ and H-4′, and H-5′ and H-7′ are at 7.04 ppm and 6.73 ppm, respectively. In hydroxylated OLC, the protons are not symmetrical and appear at 6.66 ppm (H-4′), 6.71 ppm (H-7′) and 6.55 ppm (H-8′). The signals corresponding to H-4 ’and H-7′ protons may overlap with our signals, but not that of H-8′, and the absence of a signal at 6.55 ppm indicates the OLC was not hydroxylated. The protons of aldehydes are also of diagnostic value. The protons of hydroxylated OLC are described as appearing at 9.19 and 9.54 ppm in acetonitrile, which despite being very close to our signals (just 0.02 ppm away) should be distinguishable [1].

With respect to hemiacetal and acetal, a study carried out in 2017 [2] showed that the use of methanol–water solutions for the extraction of phenols (including OLC) did not influence their formation. In addition, the quantitative responses led to the conclusion that acetonitrile and methanol–water had a similar extraction efficiency for phenols in virgin olive oils. Moreover, very low concentrations of acetals and hemiacetals from OLC were only detected when methanol gradients under acidic conditions were used for chromatographic separation. Methanol has also previously been used in solid–liquid extraction of biological fluids, gastrointestinal contents and metabolic tissues to study differences in the absorption and metabolism of hydroxytyrosol, either in its free form or through its naturally occurring esterified precursors, oleuropein and oleacein [3], and in biological samples after the ingestion of a phenolic extract from olive cake (including OLC) [4,5].

**Figure 2:**
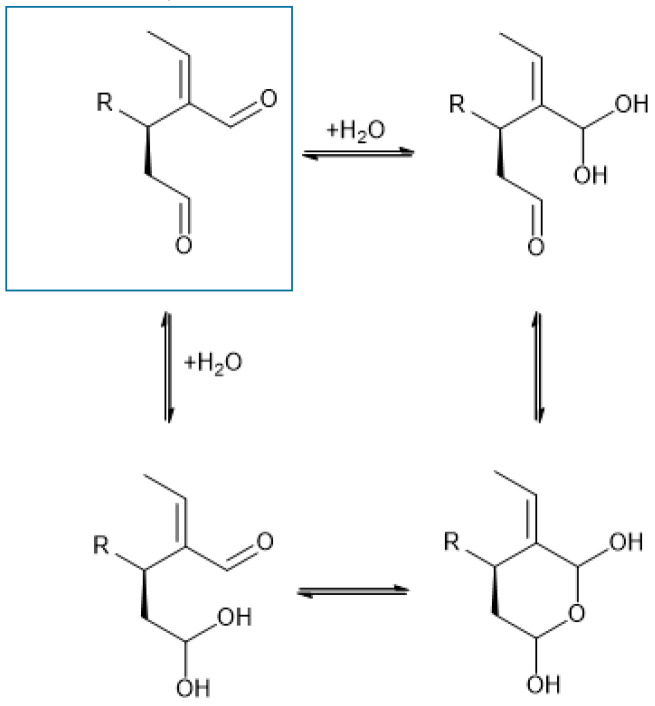
Possible cyclization to a stable pyrane ring.

Regarding metabolite B, it should be noted that we refer to *proposed* structures. Indeed, both structures are plausible. However, given that the medium (transport medium pH 7, 9.7 g/L Hanks’ Balanced Salt Solution buffered with (4-(2-hydroxyethyl)-1-piperazine ethane sulfonic acid 10 mM) is essentially neutral and includes piperazine, the structure considered wrong by A. Kaddoumi and K. El Sayed may have more potential to be the correct one (see Reference [6]). Additionally, although water addition to C=O is evidently possible, the corresponding adducts are generally thermodynamically unstable and do not accumulate. They can nevertheless act as intermediates leading to more stable structures, in this case possibly a pyrane ring via cyclization. Interestingly, both pathways converge to the same product (a bis-hemiketal) (Figure 2). Therefore, based on the medium used in the reaction, we conclude that the most likely structure is the one we propose.

Although it is true that more polar metabolites generally elute earlier than aglycones in reversed phase columns, factors other than polarity can affect elution, including molecule size and the pH. The retention time therefore reflects a compromise between different factors. Thus, while glucuronides and glucosides are more polar than the corresponding aglycone, they have a high molecular weight, and many other studies report that glucuronides elute after aglycones [5,7,8]. 

**Figure 3:**
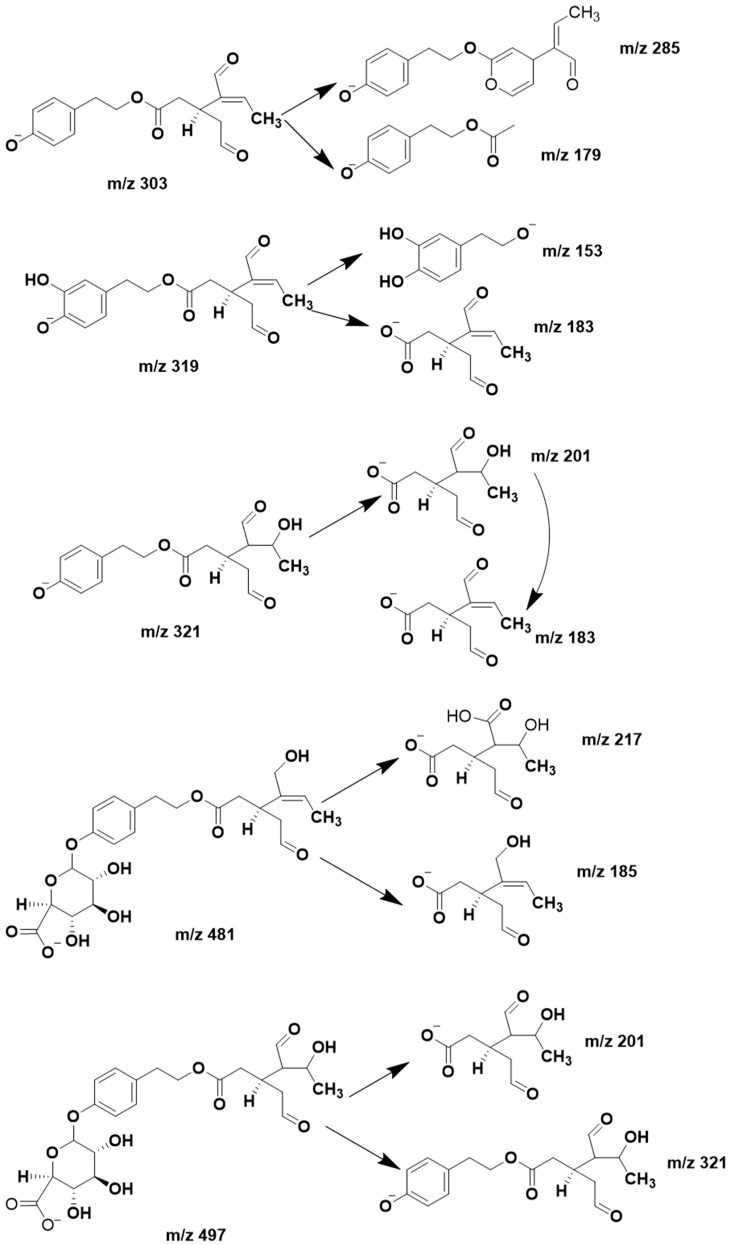
Fragments proposed for oleocanthal and its metabolites.

In their comments, A. Kaddoumi and K. El Sayed also question the fragmentation interpretation. In mass spectrometry, different equipment and conditions might result in variable fragmentation, a drawback faced by all mass spectrometrists. We did not detect the ion *m/z* 121, but nor have other studies, which report the same mass fragments of OLC as here [9,10]. Figure 3 shows the fragments that we have postulated for each compound.

A stability study of OLC in blood at 37 °C for 5 min, which is the time that OLC was in contact with blood in each sample, was not performed. However, we did not detect any OLC hydrolysis products in plasma during this time. It should be mentioned that the blood was kept in ice slush immediately after collection, and then centrifuged at 4 °C for 5 min and stored at −80 °C. Thus, either these conditions were not the most favorable for blood esterase activity, or hydrolysis did not occur, at least not in our experimental conditions (the evaluation could have been carried out in the intestine, where esterases are also distributed) [11]. OLC incubation in blood at 37 °C for a longer time (1 h) could provide more information about the potential hydrolysis mentioned in the comments, as well as about a possible distribution to erythrocytes, and will be taken into account in future studies. 

Finally, our aim was not molecular elucidation but to study the intestinal absorption and metabolism of OLC using an in situ perfusion technique in rats. Due to its complexity and diversity, successful structural elucidation requires an array of complementary analytical technologies. Furthermore, the isolation of these metabolites from plasma is difficult, resulting in only small quantities. In the literature, there are no NMR studies of OLC in blood and urine, since the concentrations in biological samples are too low for this technology.

### 2.2. Perfusion Technique

Equation (1) of the paper contains a typing error: the negative sign should be removed. However, the equation used for the calculations is correct (confirmed after reviewing the spreadsheets and calculations). Correctly written, Equation (1) is:

(1) $$P_{\mathrm{eff}}=\frac{{Ø}_{\mathrm{in}}}{2\pi RL}\times Ln\;\frac{C_{\mathrm{in}}}{C_{\mathrm{out}.\mathrm{cor}}}\;\;\;\;\;\;\mathrm{or}\;\;\;\;\;\;\;P_{\mathrm{eff}}=\frac{-{Ø}_{\mathrm{in}}}{2\pi RL}\times Ln\;\frac{C_{\mathrm{out}.\mathrm{cor}}}{C_{\mathrm{in}}}$$

The equation used to correct the OLC concentration in the intestinal lumen (Equation (2)) is also correct [12,13,14,15,16].

(2) $$C_{\mathrm{out}.\mathrm{cor}}=C_{\mathrm{out}}\times \frac{CPR_{\mathrm{in}}}{CPR_{\mathrm{out}}}$$

Roos et al. 2017 [17,18], based on the work of Sutton and Rinaldi [19], use the same equation but adding the correction factor of 1.15 (Equation (3)):

(3) $$C_{\mathrm{out}.\mathrm{cor}}=C_{\mathrm{out}}\times \frac{CPR_{\mathrm{in}}}{CPR_{\mathrm{out}}}\times 1.15$$

Sutton and Rinaldi carried out a comparative study of gravimetric, phenol red (PR) and ^14^C-PEG4000-3350 methods to determine water absorption in the rat single-pass intestinal perfusion (SPIP) model. They concluded that the gravimetric method for correcting water flux is as accurate as the two “non-absorbed” marker methods. They also reported that the average water flux (WF) for 10 compounds by either gravimetric or PR methods was 75.3 and 65.41 mL/h/cm, respectively, giving a WF_grav_/WF_PR_ ratio = 1.15 (correction factor of Equation (3)). However, this correction factor was obtained only for n = 3 animal preparations per compound and showed high variability. As an illustration, when using the PR method, the values of WF range between −21.7 and 133.8 mL/h/cm. Omitting the negative value of −21.7 (the only negative value among all the data), the ratio changes to 0.987. Thus, the correction factor of 1.15 is very sensitive, and its statistical confirmation would require more data. Tuğcu Demiröz et al. [20] also found a slightly lower estimation of the water reabsorption coefficient with the PR method, but this difference did not affect the estimation of the absorption rate coefficient of the assayed drugs. Therefore, in our opinion, Equations (2) and (3), both of which are currently being used by numerous researchers, are correct, and the lower than expected levofloxacin (LEV) permeability has other explanations (some noted in the paper). As far as we know, only one study has been published on the intestinal permeability of LEV in rats, which unfortunately is available only in Chinese [21]. The study of Volpe D.A. concerning to permeability of fluoroquinolones and cited in our paper [22] was carried out in a different model (*in vitro* and not *in vivo*; static vs. perfusion model), and different physiological factors can influence the results. To obtain further information, LEV and OLC should be tested *in vitro* on Caco-2 cells under the same experimental conditions, as interlaboratory variability is also possible. Accordingly, the results for LEV cannot be considered invalid and/or inconsistent. In addition, further investigation of LEV permeability in the SPIP model in rats would be the subject of another study and was not the aim of the present work. In our study, the highly permeable LEV was used only as a reference and for comparative purposes. Nevertheless, it would have been interesting to include a second reference standard (of low permeability).

The terms “amphiphilia” and “lipophilia” are not contradictory and express different molecular properties. In the context of our work, in which the permeability of a compound was studied, lipophilia is more appropriate, being a good predictor of intestinal permeability [23]. A common measure of the lipophilicity of a substance is its partitioning between n-octanol and water [24], and a hyperbolic relationship between lipophilia, expressed as logP, and intestinal absorption is described [25]. Such relationships are very important. OLC has a logarithm of the partition coefficient (logP) of 1.15 (similar to that of metoprolol log, P = 1.73), that is, a lipophilic property, which together with its low molecular weight, constitute favorable properties for its transport through the intestinal membrane by passive mechanisms. For this reason, OLC is considered to have a relatively high lipophilicity.

On the other hand, regarding OLC solubility, Qusa et al. 2019 [26] and Siddique et al. 2019 [27], reported a solubility of 400–500 µg/mL, which according to Wolk et al. 2014 [23] indicates that OLC is “very slightly soluble”. Accordingly, there is no contradiction. We agree with A. Kaddoumi and K. El Sayed that OLC is soluble in water at the studied concentration (0.1 mg/mL). In fact, this is a requirement for the methodology used in the intestinal permeability study and guarantees a correct determination of intestinal permeability without solubility limitations. In the “Results and Discussion” section, the following statement appears: “According to its physicochemical properties (low solubility and relatively high lipophilicity), this compound meets the characteristics of BCS class 2”. This statement is based on the physicochemical properties of OLC, and while there is no contradiction regarding its solubility and lipophilicity (bibliographic data), from a regulatory point of view, there is insufficient information to affirm that OLC belongs to class 2. Certainly, the solubility should be determined at 37 °C, within the range of physiological pH of the intestinal tract (pH 1.2-4.5-6.8) and also at the dose number [28,29]. However, it must be noted that such determinations are currently impracticable due to the scarcity of the product and suppliers, and its high cost. The aforementioned biopharmaceutics classification (BCS) based on solubility and lipophilic properties was only “provisional” and without regulatory pretensions. In the context of the present work and state of the art of OLC research, the intention was to enrich the discussion and explain the obtained permeability results. 

Elsewhere in their comments, A. Kaddoumi and K. El Sayed question how the samples were collected, focusing on the statement, “the outflow perfusate was collected in 1.5 mL amber vials at 5 min intervals for 60 min”. We agree that the sample collection should be described in more detail. To be more precise, the output perfusate was collected in *several* 1.5 mL amber vials because at the time of conducting the experiment, and to avoid isomerization at that moment, we only had available 1.5 mL amber Eppendorfs to take to the animal facilities laboratory. We realize that it would have been more practical to collect the samples directly in 5-mL amber tubes, but these were unavailable at the time. The remaining sample manipulation was done in our laboratory under infrared light conditions: the aliquots were centrifuged (7516 g for 10 min at 4 °C), the supernatant was transferred to 5-mL volume tubes and mixed, and the samples were stored at −80 °C until analysis. We have acquired 5-mL amber tubes for future experiments.

The perfusion flow rate used in the study (1 mL/min), while not the most frequent, has been used by other authors [30]. We agree that this factor can affect the intestinal residence time of the compounds tested and their absorption. However, to facilitate the interpretation of the obtained results, a parallel study was conducted with a highly permeable compound, tested under the same experimental conditions.

Finally, in the conclusions, we noted, “previous research has indicated that higher levels of OLC reach human plasma than in rats [11]”, taken from Lenerma’s study [31]. In this preclinical study, we did not provide human data, and with this statement, we only wished to refer to the fact that permeability values are usually higher in humans than in rats. We apologize if the wording is insufficiently clear.

## 3. Conclusions

We would like to conclude this reply with the following remarks: (i) OLC is a very interesting and promising constituent of extra virgin olive oil in terms of health and is still underexplored; (ii) this is the first in vivo study of OLC in rats, which (iii) can serve as a starting point for future research. We hope that the scientific community will benefit from this study and the comments it has generated.
